# Hanhnemann Club of Toronto

**Published:** 1888-05

**Authors:** J. D. Tyrrell

**Affiliations:** Secretary-Treasurer


### HAHNEMANN CLUB OF TORONTO.

Regular meeting of the Hahnemann Club opened at half-past eight P. M. The papers on convulsions were read as follows: Dr. Hall read a letter from William Young, Esq., in the *Homœopathic World*, of Oct. '87, page 472, concerning a one thousand pound reward offered by The Grocers' Company for "a method by which the vaccine contagium may be cultivated apart from the animal body in some medium or media not otherwise zymotic, etc." Mr. Young contends that the question is not an open one but is already solved by an "artificial vaccine lymph" (?), tartarized antimony in glycerine, glycerine and water, or other suitable medium. The point I wish to take exception to is vaccination with lymph from pustules produced by tartarized antimony on the ground that "such vaccination is perfectly harmless." There is not the slightest ground, to my mind, for assuming that tartarized antimony can operate in and through the animal economy to produce lymph and pustules without such lymph being influenced injuriously by the morbid conditions of the animal just as much as vaccine lymph; to be freed from *such* influences and harmless in propagating disease tartarized antimony in solution should be used immediately, not mediately. Lack of space prevents me entering more fully on this subject at this present time. Post-partum hemorrhage will occupy our attention at our next meeting, remedies assigned in groups as follows: Acon., Caul., Erigeron, Dr. Evans; Arg-nit., Kreos., Carbo veg., Dr. Hall; Cham., Nitr-ac., Apis, Dr. Tyrrell; Arnica, Apocyn., Bry., Dr. Emory; Plat., Sulf-ac., Stram., Dr. Eadie, and Ham., Hyosc., Helonias, Dr. Adams.

J. D. TYRRELL, *Secretary-Treasurer.*

Dr. John Hall, Sr., said: "In virtue of the position which my colleagues have kindly placed me in, I may be allowed a few words on our Club, and, having practiced in Toronto now some thirty-one years without such a concentration of true men to consult with, and when my cases were often met by ridicule, even from those who called themselves the followers of Hahnemann, I may say that this Club—formed as it is of those only who are sincere in their professions—promises the greatest possible good to our cause, for it is well known by our brethren of the old school of medicine that not a few who sail under our flag calling themselves homœopaths do not hesitate every now and then to fall back on various expedients for the so-called cure of disease or relief from pain which we do not acknowledge any more than those who do such things, and this being known as also by not a few of the intelligent laity, it becomes us to keep our practice pure, conforming in everything to those principles which we have espoused, that all men may see that there is a wide difference between those who practice Homœopathy and those who merely ride the homœopathic horse into position, ever using that abominable and often answered argument that it is lawful to do *anything* which may benefit our patient, an argument based on ignorance and conceived in prejudice, assuming that the true homœopath does not care as much for his patient's recovery as others.

"There is already a strong movement in England in favor of a *partial truth* in our theory, that the principle, 'similia similbus curantur'—likes are best treated by likes—is acknowledged by eminent medical men as containing a *grain of truth,* carefully ignoring, however, that Hahnemann elucidated this truth and showed its *universal application.* Passing by him altogether to the old Greek Hippocrates, who first originated the doctrine and which Hahnemann was the earliest to show, these great men have at last discovered that Hippocrates was right, beginning to acknowledge that what he very patiently taught as true is correct. But the great principle of its universal application in the treatment of ALL non-surgical diseases, a doctrine which *Hahnemann alone enunciated* and for which all who know him will forever bless his name, is carefully ignored, even as though it had never existed. Our duty then is to be very thankful for small favors. I wait for more, which will surely come if the members of this Club are true to their principles. I may therefore ask my colleagues that our union may always be our *first* consideration, setting aside all *personal differences* which will arise in its favor, that the Hahnemann Club of Toronto may not only increase in its membership, but spread its benign influence all over this Dominion and to those countries who read our papers."

---

### BELLADONNA.

When Hahnemann was compelled by the persecutions he endured to leave Leipsic, poverty in the meantime staring him in the face, he found an occupation for his skill in the employment of one of the German princes, and while there, being only partially engaged and never idle, he employed his great mind on the *causes of chronic diseases*, that is, why do men suffer from those diseases which are often brought with them into the world and over which the most rigid regimen, though keeping them in subjection, fails to make a cure or a total eradication? We may thank God, the secret disposer of all events, that Hahnemann was thus driven out and so occupied, otherwise we might never have had his *Chronic Diseases*, from which we see, as wisely said by our poet:

> "There is a divinity which shapes our ends
> Rough hew them as we will."

The subject which has fallen to my lot to write upon is one which of all others shows the presence of a dyscrasia, often inherited and sometimes caused or brought into activity by bad habits. The object of the physician is to detect this morbid condition, and when he can do that, and prescribe for the totality of the symptoms, agreeably to our rule, that "Likes are *only* cured by likes," he will surely succeed in all those cases where a return to life and health are possible. To aid in this work is the object of this paper. *First*, then, let me observe that *Belladonna* is suitable mainly in those diseases which *come on suddenly* and *as quickly depart*, and though convulsion may not always come with this list, still where we have no *preceding* illness, that fact alone will bring the remedy prominently before us.

2. This medicine is almost always a *chilly one*, the patient being *very subject to draughts of air* and liable easily to take cold from them or *having the hair cut*, also *after riding* in *a cold wind* (Aconite), the *tonsils swell, preventing deglutition.*

3. Another symptom we do well to notice, the pains are

*almost always in short attacks*, from which we may infer that even the convulsions will not generally last long.

4. *Eclampsia*, by which is meant those convulsions characterized by a flash of light before the fit, whether such be in children or adults, *especially children*. Head usually hot, *twitchings*, more in the arms and face, difficult articulation, *throws the head back*, rolling of the head. *Becomes unconscious when working in the hot sun* (Glonoine); jaws fixed, head hot, feet cold, *spasm one side* and *paralysis of the other*.

*Tetanus* and *trismus*, *epileptic convulsions* followed by *apoplexy or not*, *convulsions commence in the arm*, spasmodic motions of the body, *generally backward;* constant change from emprosthotonus to opisthotonus; *bodily inquietude*, with spasm repeated, turning the body to and fro, especially the hands and feet.

*Hysteria* in which *headache* or heat of the head predominates, worse when awaking out of sleep, also of the flexor muscles.

John Hall, Sr., M. D.

---

### CICUTA VIROSA.

In confining ourselves to the consideration of the indications for various remedies in emergency cases of convulsions, it would be unwise to cite all the symptoms produced by this drug, which might have a bearing on any form of convulsions, for they would make a volume so large that it would tax the best memory to remember them. I shall, therefore, confine myself in these remarks to the most salient features contained in the pathogenesis of this almost peerless remedy in its anti-spasmodic capacity.

In general Cicuta acts particularly upon the nervous system; *is a cerebro-spinal irritant, producing tetanus, epileptic and epileptiform convulsions, trismus, and local and general tonic and clonic spasms.*

The face may be deathly pale, drawn, cold, and expressionless, or the countenance may be hideous, with rolling eyes and piercing cries, or the face may be red or bluish, puffed up. The pallor and cold face may be preceded by flushed face and restless sleep, with headache.

Convulsions of facial muscles; distortions either horrible or ridiculous.

Eyes staring, fixed, and glassy, or upturned eyes.

Pupils contracted in spasmodic affections or contract and dilate alternately, at intervals. Trembling or twitching of eyelids.

Strabismus convergens of children is spasmodic in character or caused by convulsions.

Lockjaw, with teeth pressing firmly against one another.

Grinding of teeth, with pressing of jaws together like lockjaw.

No control over movements of mouth or tongue, preventing speaking.

Swelling of tongue, bites his tongue.

Foam in and at the mouth, unquenchable thirst, drinks swallowed with difficulty. Sudden shock deep in pit of stomach causes opisthotonus.

She feels a blow deep in the epigastrium, which passes like lightning up the back and causes her to throw herself backward in the midst of most tormenting pains.

### GENERAL TONIC SPASMS DURING CATAMENIA.

Spasms during parturition; convulsions continue after delivery, very violent and exhausting; frequent interruptions of breathing; after respiration has been restored patient remains weak and insensible, as if dead. Puerperal spasms, with trembling of limbs. Jerks as from electric shocks, borborygmus, want of breath, opisthotonus, cold, pale face, half closed eyes, with blue margins.

Great dyspnœa arising from tonic spasms of pectoral muscles.

Pulse weak, slow, or rapid, at times imperceptible.

Violent tonic spasms of pectoral muscles. The muscles of neck contract and become hard as wood.

Spasmodic drawing of the head backward.

Tetanic stiffness of back.

Spasmodic jerkings of arms and upper part of body following injury to knee by splinter.

Jerking in arms, left arm all day.

Tonic spasms of arms, fingers clenched, thumbs turned inward.

Spasmodic contortions and fearful jerkings of all the limbs, with violent convulsions.

State of insensibility and immobility, with loss of strength and consciousness.

Catalepsy, limbs hang down, and patient appears lifeless.

Dysphagia; no muscular flinching when trunk or lower extremities were pricked with a pin, perfect paralysis, not moving or winking for several days. Cerebro-spinal meningitis.

Sudden rigidity, with jerks, afterward great relaxation and weakness.

Strange contortions of upper part of body and limbs during paroxysms, with blue face and frequent interruptions of breathing for a few moments.

Child suddenly grasped knee with his hands and screamed fearfully; immediately afterward was seized with convulsions and insensibility, head being permanently retracted.

Convulsions with frightful contortions and distortions of limbs and body, loss of consciousness, high fever, vomiting, dilated pupils, double vision, ashy paleness, one diarrhœic stool, then constipation.

Convulsions with limbs relaxed and hanging down or unnaturally stiffened and extended. Epilepsy, with violent contortions of limbs and upper part of body and head, bluish face, interrupted respiration, and frothing at the mouth.

Epileptic attacks, with swelling of stomach, as from violent spasms of diaphragm; hiccough, screaming; redness of face; trismus; loss of consciousness and distortions of limbs.

*Violent tonic spasms of any or all the muscles, each muscle being perfectly rigid, so that neither the curved limbs can be straightened out nor the straight ones curved. Tonic spasms renewed from slightest touch, from opening of door, or loud talking.*

Attacks come on twice a day, and she seems dying in them. Periodic ecstasy. Child lies on back, not being able to turn, legs alternately contracted and extended.

After convulsions child is unconscious and nearly lifeless.

She thinks she sees coming toward her a huge drunken man, who lies down beside her; she begs him to retire, falls into convulsions, and turns over on her abdomen.

He thinks himself a young child again, confounds the present with the past, everything seems strange, as in a strange place, with fear.

W. J. HUNTER EMORY, M. D.

---

### CINA.

From a drug causing such a marked and continuous irritation of the system in general and nervous system in particular, we would naturally expect that CINA would be frequently called for in the treatment of convulsions and spasmodic affections generally.

Neither will such expectations be disappointed, though more commonly useful in children or the weak and debilitated—in short, the anæmic—in cases where a primary irritation produces a reflex nervous or sympathetic influence upon the great nerve centres. Take as examples of my meaning the spasmodic diseases of children affected with worms, or infants suffering from a pathological teething process.

In fact, the pathogenesy of this drug is full of irritation, mentally or physically, sleeping or waking; all the symptoms and conditions the drug is capable of causing and, therefore, of curing, when caused by other means, are indicative of disquiet and unrest.

Naturally, then, we shall find when Cina is indicated that the patient is of a *whining, fretful* nature, *irritable in himself*, and irritating to all around. Complaining, restless asleep or awake, requiring constant attention, yet objecting to any one *touching, speaking to*, or *coming near* him. This being the mental state, you can readily see that convulsive or spasmodic conditions are not distant.

Briefly, I will try to collate the prominent and characteristic symptoms, which, when hurriedly called to a patient suffering from convulsions, would lead us prescribe *Cina* as the simillimum.

A weak, hollow, empty feeling in the head—in general a stupefying, dull, drawing pain in the head.

Headache *before and after* epileptic attacks.

Headache and general aggravation from *steadily gazing at one object*, as in sewing.

Intermitting pressure, as from a heavy weight, as if the brain was pressed down from middle of vertex.

The face presents a pale, sickly appearance, is cold or red, or *one cheek red, the other pale.* [Compare *Cham.*, in which the *left cheek is red, the right pale.*] In short, the face as a rule expresses intense misery.

In the eyes the pupils commonly dilated; eyeballs convulsively turned upward, showing only the whites, or fixed and staring, with dilated pupils; eyes staring, or a drooping and heaviness of the lids; optical illusions in bright colors—*strabismus.*

*Boring in, picking at, or rubbing of the nose.*

Strabismus caused by the irritation of worms.

*Cylindrical contraction of the tongue, which is spasmodically forced through the lips.*

Choreic movements extend to tongue, œsophagus, and larynx, causing a clucking noise from throat to stomach.

*Frequent motion in throat as though swallowing something*; specially noticeable after a spell of coughing (hacking).

Frequent hiccough, also *during sleep.*

Dry, spasmodic cough, with rigidity of the body and unconsciousness. *Before coughing childs rises suddenly, looks wildly*

*around, the whole body becomes stiff, she loses consciousness, just as though she would have an epileptic attack, then follows the cough.*

*Oversensitiveness of the body to touch.*

Spasmodic yawning, pains removed by yawning, which cannot be suppressed.

*Twitching, jerking, and distortion of the limbs.*

General rigor of the body, with loss of consciousness. Spasms of children, with throwing the arms from side to side. *Convulsive attacks at night.*

Convulsions of extensor muscles; *sudden stiffness, backward;* a clucking noise, as though water was poured from a bottle, from throat to abdomen.

Epileptic attacks, *specially at night.* Epileptiform convulsions, with consciousness.

Eclampsia, specially at night, with or without loss of consciousness; lying on the back; violent screams and violent jerks of hands and feet.

*Chorea from intestinal irritation. Sudden distressing cries in sleep; grinding teeth during sleep; awakes from sleep with trembling and marked signs of fear, with screams and cannot be pacified.* Child will not sleep unless rocked or kept in constant motion. *Child hangs its head to one side,* is drowsy. Verminal spasms in children are preceded by a chill, are accompanied by high fever; heat mostly in head and face; pain in stomach and abdomen; *pupils dilated; restlessness, with grinding of the teeth; sudden starts;* abdomen bloated.

The left leg is flexed both on itself and on the abdomen.

In attacks *with loss of consciousness,* we find *frothing from the mouth.*

Reflex spasms in the dominion of the nervus vagus. *Milky urine.*

*Ravenous hunger,* even after severe vomiting, wants to eat again.

Aggravation from *external pressure; at night; on looking at an object steadily.* Also from exposure to the open air; from cold air; from cold water; generally *from cold.* EDWARD ADAMS.

---

### CHAMOMILLA IN PUERPERAL AND OTHER CONVULSIONS.

The cases calling for the use of this drug are those in which the patient either cannot or will not exercise proper control over the functions of the mind or body. Owing to a similar want of equilibrium, the system generally is affected to an immoderate extent by local irritation of various parts, and there is a decided lack of balance in the effect on the two sides of the body, the left side generally suffering more than the right.

It is indicated in convulsions caused by giving way to a fit of passion, as well as in those occurrences during difficult dentition, where the gums are red and tender.

The attacks are violent; the child screams immoderately; stiffens and bends backward; kicks and moves the legs up and down; grasps and reaches with the hands, and draws the corners of the mouth alternately to one side and the other, while twitchings of the eyelids and grimaces of the face in general help to make complete the picture of a naughty, naughty child who does not even so much as desire to be good, but, perhaps, with one-sided flush of face, screams for things which it does not want, and utterly refuses to come to terms except on the condition, severe enough on the wearied nurse, of being carried up and down the room.

It would seem from the above sketch to be especially suitable to children, or to those who, though grown to years of discretion, yet retain some of the less attractive traits of childhood.

L. Hamilton Evans, M. D.

---

### ACONITE.

Aconite produces a condition of oversensitiveness of the body to external impressions, and a painful sensitiveness of the body to contact; cannot bear light or noise; will not be touched; this is a condition related to that found in convulsions, due to reflex excitability, such as those induced by the presence of foreign bodies in the canals, to indigestion, or to constipation.

The Aconite state is marked by inconsolable anxiety, loud complaining, restless, agonized tossing about; believes death near, and is afraid of it. Aconite should be thought of in convulsions of children, due to anger or paroxysms of rage, and to fright, also in teething, with heat, startings, and twitching of single muscles; child gnaws its fists, frets, and cries.

Aconite has retention and suppression of urine, and will be found of service in acute uræmic convulsions, when characterized by the peculiar Aconite morale, possibly in the puerperal forms and the acute inflammatory varieties. Where at this late date rational medicine hesitates not to prescribe venesection. In convulsions of children with retention of urine, especially when accompanied by restlessness and fear, with tossing about.

In summer diarrhœa with convulsions, Aconite will often be of service. Stool like chopped herbs; with violent pains in the bowels; from getting wet or occurring during hot days and cold nights. Aconite attacks are likely to occur at night and after exposure to dry, cold winds. It is often indicated in convulsive or spasmodic affections of the respiratory organs; in croup, awaking in first sleep; child in agony; impatient, tossing about; grasps its throat every time it coughs in great distress and fear.

In children pneumonia of the apex of the lung is often attended with convulsions at the outset, in which Aconite may be called for. Hahnemann says, "Every time when Aconite is chosen as a homœopathic remedy, it is especially necessary to regard the moral symptoms, and be careful that they resemble those that belong to it."

So universally are the mental symptoms present in cases requiring Aconite, that it may be safely rejected in cases marked by a quiet morale. A. B. Eadie.

---

### STRAMONIUM.

Having, in common with others, taken upon ourselves the honorable name of Homœopathy, we thereby proclaim to the public our convictions of the existence and truth of the natural law of cure, "Similia similibus curantur." We, therefore, stand pledged to suffering humanity to maintain an unswerving allegiance to our mistress, to keep ourselves pure and undefiled, and to faithfully practice the principles we profess. Failing in this, we stand before the world in the lamentable position of self-convicted hypocrisy.

If we deviate from the path of homœopathic rectitude and pursue that *ignus fatuus*, "broad-minded liberality," we shall soon find ourselves sinking in the swamp of mongrelism, our robes covered with its muck and mire, and ourselves an abhorrence alike to friend and foe.

While it is *not* in our power to command the *love* of the dominant school, we can so live and practice as to compel all men to respect us. Speaking of Homœopathy, an eminent allopath of New York once said: "I can respect those of your profession who adhere in their practice to the doctrines you teach, but for the mongrels among you, and they are many, who always use crude drugs, and in difficult cases resort to our remedies, I can only regard them as bad men, and must hold them in utter contempt." Let us put on the whole armor and quit ourselves like men, and if, through ignorance or weakness, we stumble in the way, or even fall to earth, let us, like Antæus, gather fresh strength for the conflict.

Trusting you will pardon this brief exordium, I now address myself to the remedy assigned me and the subject in hand—Stramonium in convulsions. This remedy is so rich in symptoms pertaining to convulsive seizures, that one cannot, in a short paper, do it justice, and my *own weakness* is a bar sinister in *any* paper. It is eminently adapted to convulsions, epileptic fits, chorea, catalepsy, and other spasmodic affections, especially from fright, and inhaling vapor of Mercury; convulsive movements or jerks, especially when touched, or from fixing eyes on brilliant objects, such as a light, mirror, or water, or else coming on periodically; contraction and slow extension of the limbs; tetanus, opisthotonus; attacks of cramp; convulsive jerking of limbs, with weeping.

Hysterical convulsions; rigid, nervous chills; frothing at mouth; faces appear elongated; on first seeing any one, she shrinks back with fear; tries to escape. Convulsions as in epilepsy, but without loss of consciousness; from fright; attacks sudden, with screams, afterward drowsy but cannot sleep; attacks are periodical, and there are premonitory symptoms. Tonic spasms, as catalepsy, limbs can be moved by others; stiffness of body, with loss of consciousness, preceded by headache, with vertigo.

In apoplectic seizures we will find this a valuable remedy; paroxysms of syncope, with stertorous respirations; deep sleep, with snoring and a bloody froth at mouth; dark brown face; he lies on back with open, staring eyes; fetches breath with great difficulty. *Paralysis*, also after *apoplexy*, spasmodic drawing of head to either side; with screaming, with convulsive movements of arms above head, especially in eclampsia.

Convulsions of head and arms, with hiccough; moves head to and fro, this movement is only interrupted by the hiccough. Hydrophobia; water, mirror, or any bright object excites convulsions; restless, violent, violent convulsions; screams, bites; mouth dry; delirious, without memory or consciousness; pupils extremely dilated; violent desire to bite and tear everything with his teeth; eyes wide open, staring, brilliant; wild and red. Alternation of convulsions and rage; spasms so violent he could scarcely be held, as spasms abate became furious and beat and bit those who held him (*cf.* Hyosc., Bell., Canth., and Lyssin). Vehement thirst, mostly with aversion to water and all fluids, shrinks from the proffered cup; even sight of water causes spasms; constriction of throat, froth, spitting; there may be violent thirst for large quantities, drinking with great avidity.

Chorea from fright; creeping in limbs, then violent movements generally crosswise; rotates arms over head; tonic and clonic spasms alternate; body very hot; continually changes character. *Mania-à-potu* (Ars.) with clonic spasms (Hyosc.), with consciousness and desire for light and company. Among the general characteristics of this drug are: Convulsions and delirium can be specially excited by contact; spasmodic symptoms of face alternate with paralytic; lock-jaw after convulsions; jerking of head clear from pillow and lets it fall again, this is continually repeated; skin of forehead wrinkled; eyes staring, glistening, and protruding; face pale, or bright red; the whole face looks wild and frightful. Dryness of mouth and throat causes dysphagia; spasmodic constriction of the pharynx. Paralysis of one, convulsions of the other side (*cf.* Artem. vulg.); arms agitated, lower limbs quiet. Cannot walk in *dark* or with *eyes shut,* will surely fall. General aggravations: After sleep (in A. M.); in solitude and in the dark (especially mental states); from touch, fright, and looking at water and glistening objects (convulsions).

Amelioration: In company and from bright light (mental symptoms); likes a well-illuminated room, though he *cannot* bear to look at the light. J. D. TYRRELL.

